# Role of CXCL10 in the progression of in situ to invasive carcinoma of the breast

**DOI:** 10.1038/s41598-021-97390-5

**Published:** 2021-09-09

**Authors:** Milim Kim, Hye Yeon Choi, Ji Won Woo, Yul Ri Chung, So Yeon Park

**Affiliations:** 1 Department of Pathology, Seoul National University Bundang Hospital, 82, Gumi-ro 173 Beon-gil, Bundang-gu, Seongnam, Gyeonggi 13620 Republic of Korea; 2 Department of Pathology, Seoul National University College of Medicine, Seoul, Republic of Korea; 3 Pathology Center, Seegene Medical Foundation, Seoul, Republic of Korea

**Keywords:** Cancer, Immunology, Oncology

## Abstract

Tumor immune microenvironment plays a crucial role in tumor progression. We performed immune profiling to compare immune-related gene expression between ductal carcinoma in situ (DCIS) and invasive carcinoma of the breast using nCounter PanCancer immune Profiling Panel and found that CXCL10 was the most significant gene that had the highest difference in expression between them. Effect of CXCL10 on breast cancer cell proliferation and invasion was examined in vitro, and expression of CXCL10 and its relationship with immune cell infiltration was assessed in breast cancer samples. CXCL10 induced cell proliferation, migration and epithelial-mesenchymal transition in MCF-7 and MDA-MB-231 breast cancer cell lines. We confirmed that CXCL10 mRNA expression was significantly higher in invasive carcinoma than in DCIS, especially in hormone receptor (HR)-negative tumors using a validation set. CXCL10 mRNA expression showed a positive correlation with tumor infiltrating lymphocyte (TIL) density in both DCIS and invasive carcinoma; CXCL10-positive tumors generally showed higher infiltration of CD8+ and FOXP3+TILs as well as PD-L1+ immune cells compared to CXCL10-negative tumors, albeit with different patterns according to HR status. In conclusion, our study showed that CXCL10 promotes tumor cell proliferation, invasion, and immune cell infiltration, implying its contribution in the progression of DCIS to invasive carcinoma of the breast.

## Introduction

Interaction between tumor cells and immune microenvironment plays a critical role in tumor development and progression^1^. Key players in tumor immune microenvironment include various types of myeloid cells, lymphocytes, cytokines, and chemokines, and studies on tumor immunity have mainly focused on immune cells and their interaction with the tumor^2,3^. However, growing evidence suggests that other factors such as chemokines are known to be actively involved in tumor progression.

Chemokines are small proteins, usually between 8 and 10 kDa, that provide leukocytes with directional cues for development, homeostasis, and inflammation through interaction with a subset of seven-transmembrane G protein-coupled receptors (GPCRs)^4^. Their role as a pro-inflammatory mediator that attracts leukocytes at the site of inflammation is well known, and chemokines have been considered a potential target for inflammatory diseases and autoimmune diseases^5,6^. Aside from its role in inflammation, chemokines are also involved in tumor progression and metastasis through different mechanisms; cancer cell attraction to the site of metastasis, mobilization of bone marrow-derived leukocytes including regulatory T cells, myeloid derived suppressor cells, and tumor associated macrophages, and autocrine signaling for tumor growth^6^. However, in breast cancer, the role of chemokine in tumorigenesis and tumor progression remains inconclusive.

In a previous study, we evaluated the immune microenvironment of ductal carcinoma in situ (DCIS) in comparison with invasive breast cancer focusing on tumor infiltrating lymphocyte (TIL) subsets and PD-L1+ immune cells^7^. In this study, as a next step, we compared the expression of immune-related genes between DCIS and invasive carcinoma via comprehensive immune profiling. As a result, CXCL10 revealed the highest level of difference in gene expression and was selected for further analysis. We evaluated the effect of CXCL10 on tumor cell proliferation and migration using breast cancer cell lines MCF-7 and MDA-MB-231. We also evaluated whether CXCL10 induced epithelial-mesenchymal transition (EMT) in these cell lines. The difference in CXCL10 expression between DCIS and invasive carcinoma was validated by real-time polymerase chain reaction (PCR) and immunohistochemistry. Lastly, we examined the association of CXCL10 expression with TIL density and immune cell subset infiltration in DCIS and invasive carcinoma of the breast.

## Results

### Immune-related gene expression in DCIS and invasive carcinoma

Using nCounter PanCancer Immune Profiling Panels including 770 immune-related genes, the difference in immune-related gene expression was evaluated in DCIS and invasive carcinoma using the first set of samples including 16 cases of DCIS and 32 cases of invasive carcinoma. The list of top 20 immune-related genes that showed significantly different expression between DCIS and invasive carcinoma is shown in Table 1. Among the top 20 genes, those with a Log2 fold change greater than 1.5 with adjusted *p* value less than 0.05 were CXCL10 and CXCL9. Especially, CXCL10 had the greatest fold change with a Log2 fold change value of 2.92 with adjusted *p* value of 0.003. In hormone receptor (HR)-positive subgroup, none of the genes revealed a significant difference in expression between DCIS and invasive carcinoma. However, S100A8, LAG3, CXCL10, CXCL9 and BIRC5 showed a difference in fold change between DCIS and invasive carcinoma although statistically not significant (adjusted *p* value > 0.05; Supplementary Table S1). In HR-negative subgroup, no genes showed significantly different expression between DCIS and invasive carcinoma. CXCL10 had a largest fold change value of 3.44 but with adjusted *p* > 0.05. The list of top 20 genes with a difference in fold change between DCIS and invasive carcinoma in HR-negative subgroup is shown in Supplementary Table S1.

**Table 1:** List of top 20 genes with a significant fold change between DCIS and invasive carcinoma.

Gene (mRNA)	Log2 fold change	*p* value	Adjusted *p* value*
CXCL10	2.92	6.50E−07	0.003
LAG3	2.39	9.43E−05	0.114
IL32	2.35	0.00039	0.206
CXCL9	2.26	7.67E−06	0.019
PDCD1LG2	1.93	0.00018	0.145
CD96	1.82	0.00182	0.532
SH2D1A	1.81	0.00187	0.532
CD5	1.68	0.00249	0.631
TAP1	1.64	0.00128	0.476
HLA-DRA	1.55	0.00025	0.173
CCL5	1.55	0.00043	0.206
STAT1	1.41	0.00113	0.452
PLAU	1.37	1.18E−05	0.019
HLA-DPA1	1.27	0.00070	0.305
C1S	1.24	0.00018	0.145
CXCR4	0.95	0.00184	0.532
BIRC5	0.95	0.00226	0.605
CK1	0.90	0.00184	0.532
VCAM1	0.78	0.00266	0.640
CXCL2	-1.21	0.00041	0.206

*p values were adjusted by Benjamini-Yekutieli procedure.

### Comparison of CXCL10 mRNA expression in DCIS and invasive carcinoma

**Figure 1:**
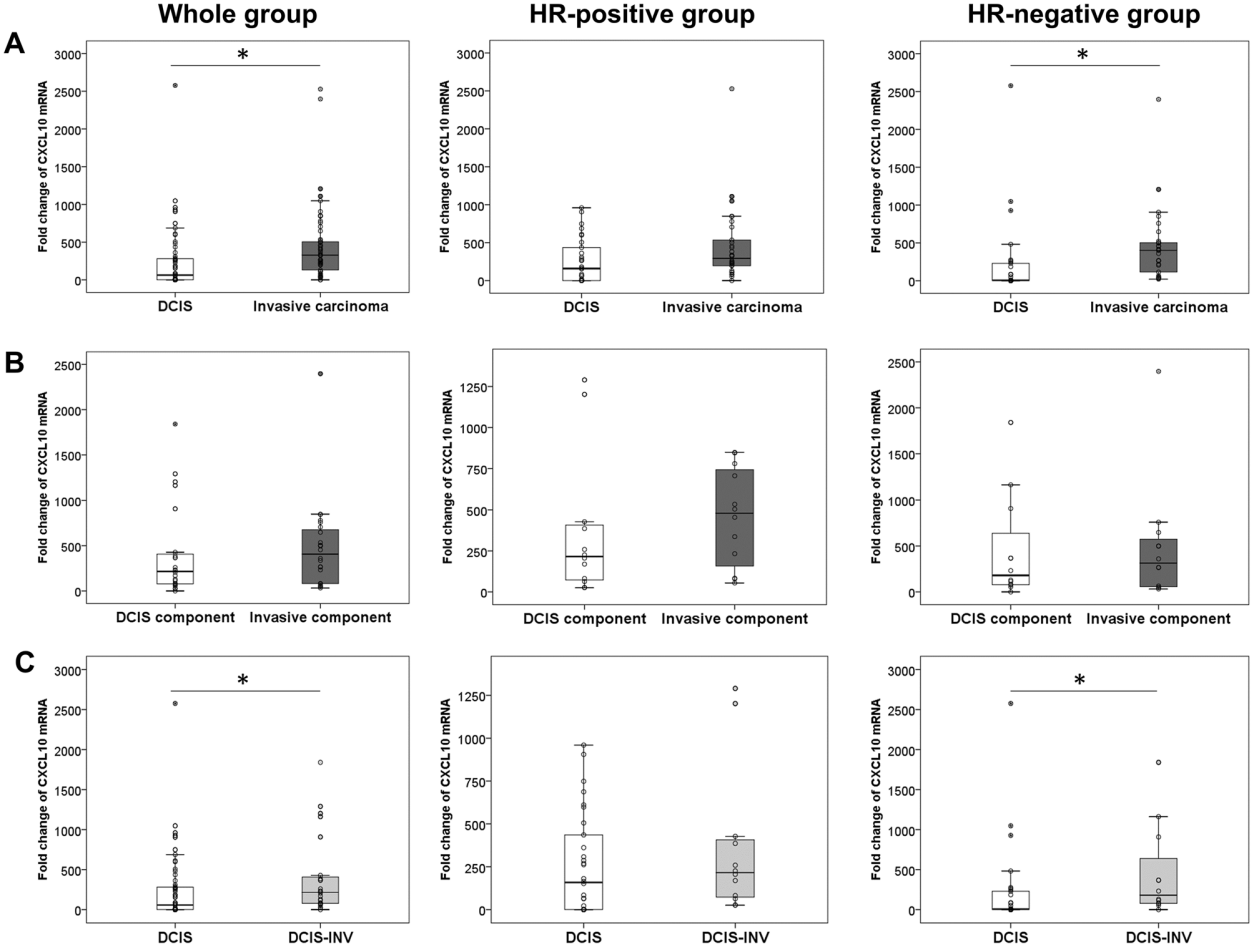
Comparison of CXCL10 mRNA expression between DCIS and invasive carcinoma. (**A**) CXCL10 mRNA expression is significantly higher in invasive carcinoma than in DCIS, in the whole group (n = 120; *p* < 0.001) and in the hormone receptor (HR)-negative group (n = 60; *p* < 0.001). (**B**) However, within individual tumors, CXCL10 mRNA expression is not significantly different between DCIS and invasive components of the same tumor in all groups (n = 24, whole group; n = 12, HR-positive and H-negative group). (**C**) In comparison of DCIS (n = 60) with DCIS associated with invasive carcinoma (DCIS-INV) (n = 24), CXCL10 mRNA expression is significantly higher in DCIS-INV than in DCIS in the whole group (n = 84; *p* = 0.011) and in HR-negative group (n = 42; *p* = 0.020). *Statistically significant. The graphs were generated using SPSS version 25.0 for Windows (IBM Corp., Armonk, NY, USA).

Using the second validation set of samples composed of 120 cases of DCIS and invasive carcinoma (60 cases in each), difference in CXCL10 mRNA expression was examined between DCIS and invasive carcinoma in the whole groups, in HR-positive subgroup, as well as in HR-negative subgroup (Fig. 1A). In the whole group, expression of CXCL10 mRNA, evaluated by the fold change (2^−ΔΔCt^), was significantly higher in invasive carcinoma than in DCIS (*p* < 0.001). However, in HR-positive subgroup, CXCL10 mRNA expression was not significantly different between the two groups (*p* = 0.260). In the HR-negative subgroup, CXCL10 mRNA expression was significantly higher in invasive carcinoma compared to DCIS (*p* < 0.001), similar to the whole group.

CXCL10 mRNA expression was also evaluated in the invasive and DCIS components within the same tumor in the 24 cases of invasive carcinoma with a sufficient DCIS component. As a whole, the invasive component of the tumors generally showed a higher level of CXCL10 mRNA expression compared to DCIS component. However, CXCL10 mRNA expression was not statistically different between DCIS and invasive components of the same tumor in the whole group, and HR-positive and HR-negative subgroups (*p* = 0.710, *p* = 0.754 and *p* = 0.875, respectively; Fig. 1B).

The difference in CXCL10 mRNA expression between DCIS and DCIS associated with invasive carcinoma (DCIS-INV) was also examined (Fig. 1C). CXCL10 mRNA expression was significantly higher in DCIS-INV than DCIS in the whole group and in HR-negative subgroup (*p* = 0.011 and *p* = 0.020, respectively). In HR-positive subgroup, there was no significant difference in CXCL10 mRNA expression between the two groups (*p* = 0.301).

### CXCL10 promotes breast cancer cell proliferation and migration

**Figure 2:**
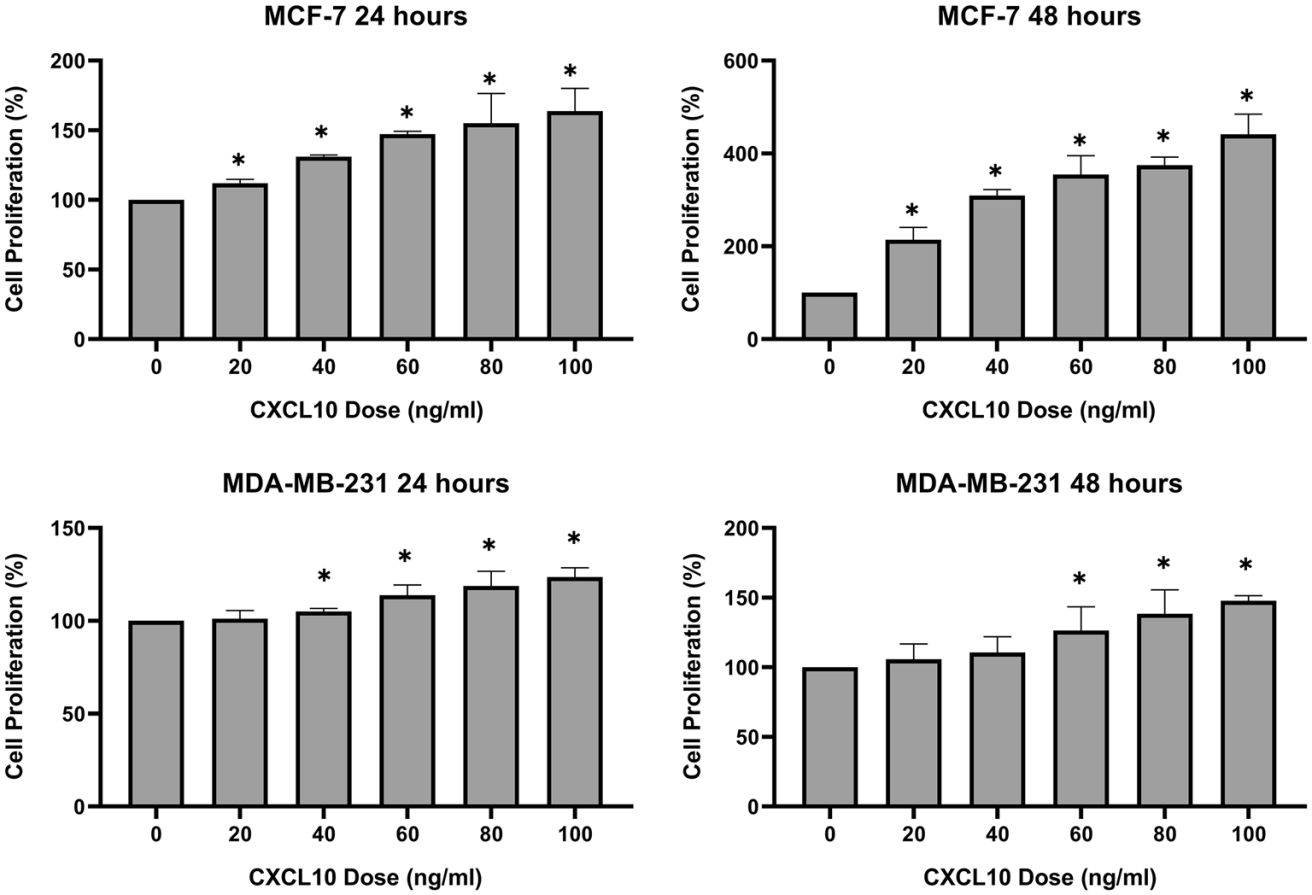
Cell proliferation with CXCL10 treatment. CXCL10 promoted cell proliferation in MCF-7 after 24 h and 48 h with a significantly increased percentage of cell proliferation in all doses added, of 20 ng/ml, 40 ng/ml, 60 ng/ml, 80 ng/ml, and 100 ng/ml, respectively (all *p* < 0.05). In MDA-MB-231, 40 ng/ml, 60 ng/ml, 80 ng/ml, and 100 ng/ml of CXCL10 significantly increased the percentage of cell proliferation after 24 h (*p* < 0.05) and 60 ng/ml, 80 ng/ml, and 100 ng/ml significantly increased cell proliferation after 48 h (*p* < 0.05). *Statistically significant. The graphs were created using GraphPad Prism version 8.02 for Windows (GraphPad Sofware, San Diego, CA, USA, www.graphpad.com).

In order to determine whether exogenous CXCL10 treatment promotes breast cancer cell proliferation, we added CXCL10 to MCF-7 and MDA-MB-231 cell lines at a concentration of 20 ng/ml, 40 ng/ml, 60 ng/ml, 80 ng/ml, and 100 ng/ml, respectively (Fig. 2). In MCF-7, cell proliferation significantly increased after 24 h and 48 h of incubation with CXCL10 in a dose-dependent manner. On the other hand, MDA-MB-231 showed cell proliferation at high concentrations of more than 60 ng/ml of CXCL10 treatment (Fig. 2).

**Figure 3:**
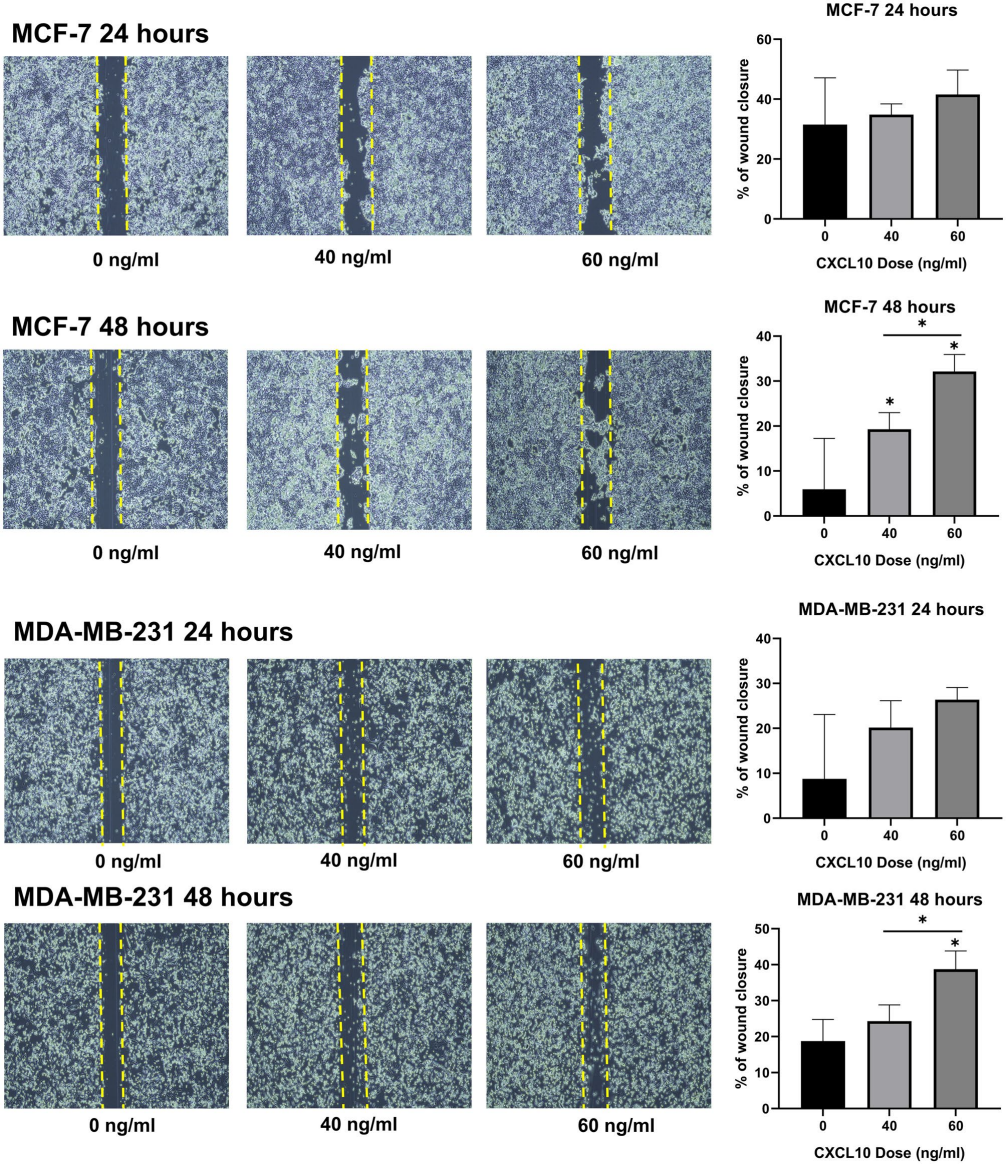
Cell migration with CXCL10 treatment. In MCF-7, cell migration increased in a stepwise manner after 24 h although statistically non-significant. After 48 h, the percentage of wound closure significantly increased with 40 ng/ml and 60 ng/ml of CXCL10 with *p* < 0.05*,* respectively. MDA-MB231 showed an increased percentage of wound closure after 24 h and 48 h after CXCL10 was added. After 48 h, the sample with 60 ng/ml of CXCL10 showed significantly increased wound closure (*p* < 0.05). *Statistically significant. The graphs were created using GraphPad Prism version 8.02 for Windows (GraphPad Sofware, San Diego, CA, USA, www.graphpad.com).

In the next step, we employed a wound healing assay to evaluate whether CXCL10 promotes breast cancer cell migration in MCF-7 and MDA-MB-231 by observing cell migration after 24 h and 48 h of CXCL10 treatment. In MCF-7, although statistically non-significant, cell migration increased in a stepwise manner after 24 h in the samples that were treated with 40 ng/ml and 60 ng/ml of CXCL10 (Fig. 3). After 48 h, the percentage of wound closure was rather accentuated, and the samples treated with both 40 ng/ml and 60 ng/ml of CXCL10 showed a significant increase in wound closure (*p* < 0.05). MDA-MB-231 showed similar results as MCF-7: migration of tumor cells in the wounded area seemed to increase when treated with CXCL10. Similar to MCF-7, after 48 h, there was a significant difference between the percentage of wound closure between 40 ng/ml of CXCL10-treated sample and 60 ng/ml of CXCL10-treated sample, with *p* < 0.05 (Fig. 3).

### CXCL10 induces epithelial-mesenchymal transition in breast cancer cells

**Figure 4:**
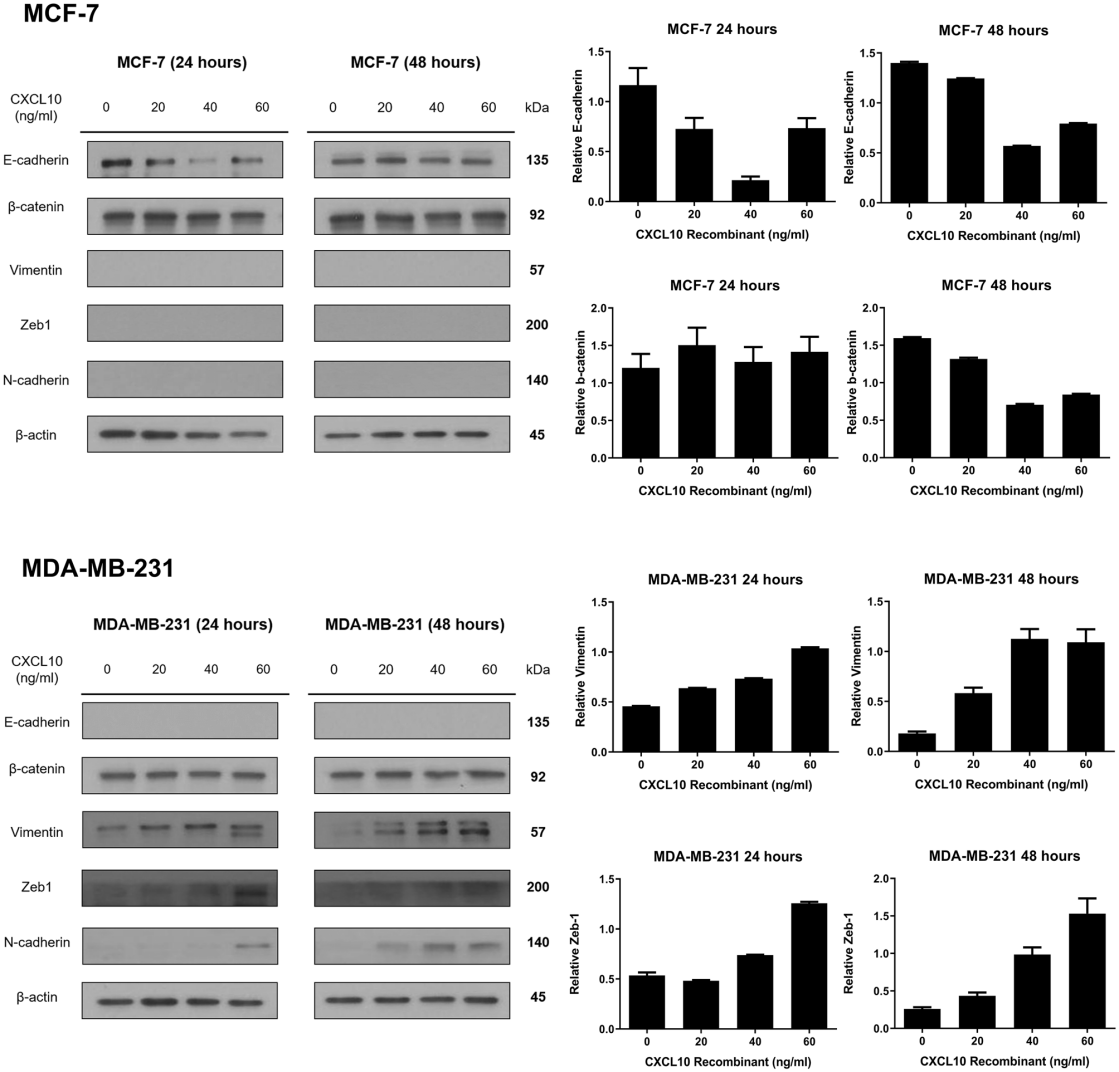
Western blot of epithelial-mesenchymal transition-related markers with CXCL10 treatment. In MCF-7, expression of E-cadherin decreased with an increased dose of CXCL10. Mesenchymal markers- β-catenin, vimentin, Zeb1 and N-cadherin—did not show any expression regardless of the dose of CXCL10 added to the sample. Western blot assay showed increased expression of vimentin, Zeb1, and N-cadherin expression with an increased dose of CXCL10 in MDA-MB-231. There was no E-cadherin expression in MDA-MB-231. *Statistically significant. The graphs were created using GraphPad Prism version 8.02 for Windows (GraphPad Sofware, San Diego, CA, USA, www.graphpad.com). Full length blots are presented in Supplementary Figure S1.

Next, we performed Western blot analysis to validate that CXCL10 does induce EMT in breast cancer. As shown in Fig. 4 and Supplementary Figure S1, CXCL10 induced decreased epithelial marker (E-cadherin) expression after 24 h, but not after 48 h in MCF-7 breast cancer cell line. β-catenin expression did not change significantly regardless of CXCL10 treatment after 24 h and slightly decreased after 48 h. Other mesenchymal markers such as vimentin, Zeb1, and N-cadherin was not expressed in MCF-7. In MDA-MB-231, expression of mesenchymal markers including vimentin, Zeb1, and N-cadherin was increased in CXCL10 treated cells in a dose-dependent manner both after 24 h and 48 h, but β-catenin expression did not show any difference in CXCL10-treated cells. There was no expression of E-cadherin in control as well as in CXCL10 treated samples.

### Correlation of CXCL10 mRNA expression with TIL infiltration in DCIS and invasive carcinoma

**Figure 5:**
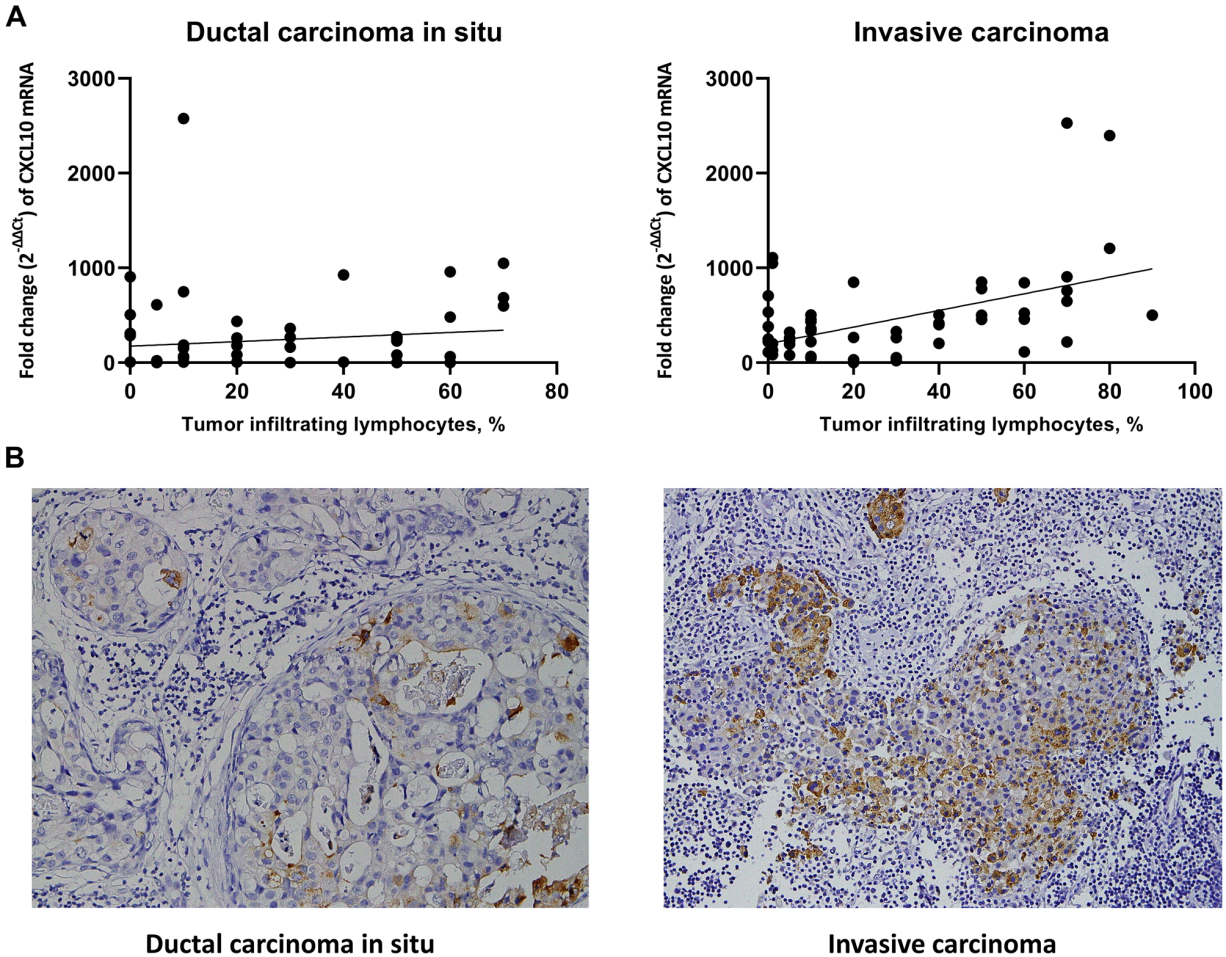
CXCL10 expression in DCIS and invasive carcinoma in relation to immune cell infiltration. (**A**) Graphs showing correlation between and CXCL10 mRNA expression and tumor infiltrating lymphocytes. In both DCIS and invasive carcinoma, tumor infiltrating lymphocyte density and CXCL10 mRNA fold change (2^−ΔΔCt^) showed a weak positive correlation, with rho = 0.270, and rho = 0.382, respectively. (**B**) Representative example of immunohistochemical expression of CXCL10 in DCIS and invasive carcinoma. Membranous or cytoplasmic expressions with a dot-like staining pattern are found in tumor cells as well as in immune cells. These cases show increased infiltrations of tumor infiltrating lymphocytes. The graphs were created using GraphPad Prism version 8.02 for Windows (GraphPad Sofware, San Diego, CA, USA, www.graphpad.com).

Since CXCL10 is a chemokine that is known to attract immune cells including T cells, the correlation between CXCL10 expression and TIL density was evaluated using the second set of samples (Fig. 5A). In DCIS, CXCL10 mRNA expression and TIL infiltration showed a weak positive correlation (rho = 0.270, *p* = 0.037). In invasive carcinoma, CXCL10 mRNA expression also showed a weak positive correlation with TIL density (rho = 0.382, *p* = 0.003).

### CXCL10 protein expression and its relationship with clinicopathologic features of tumor

CXCL10 protein expression was evaluated in the third set compose of 223 cases of DCIS and 372 cases of invasive carcinoma. In DCIS, immunohistochemistry revealed that CXCL10 expression was not frequent; however, the staining pattern was similar to that of invasive carcinoma with dot-like cytoplasmic or membranous staining in tumor cells and immune cells, especially in macrophages (Fig. 5B). Of 223 cases of DCIS, CXCL10 expression was observed in 21 cases (9.4%). In invasive carcinoma, CXCL10 expression was more commonly observed compared to DCIS with 81 cases (21.8%) of CXCL10-positive tumors among 372 cases of invasive carcinomas (*p* < 0.001). Expression of CXCL10 was mainly found in the tumor cells and immune cells although some tumor stromal cells also showed a positive staining (Fig. 5B). In HR-negative subgroup, CXCL10 expression was significantly higher in invasive carcinoma than DCIS (42.6% vs. 13.7%, *p* < 0.001). However, there was no difference between invasive carcinoma and DCIS in HR-positive subgroup (13.1% vs. 8.1%).

When evaluating clinicopathologic features of tumor in relation to CXCL10 expression, none of the clinicopathologic features were associated with CXCL10 in DCIS (Supplementary Table S3). However, in invasive carcinoma, high histologic grade, ER negativity, PR negativity, high Ki-67 proliferation index, p53 overexpression, and triple negative subtype were associated with CXCL10 expression (all *p* < 0.005; Supplementary Table S4).

### Correlation of immune cell subset infiltration with CXCL10 expression

Of the third set, 223 cases of DCIS and 151 cases of invasive carcinoma with data of immune cell infiltration were used to assess correlation of immune cell subset infiltration with CXCL10 expression. In DCIS, CXCL10 expression correlated with CD4+, CD8+, FOXP3+TIL, and PD-L1+ immune cell infiltration (Table 2). In the whole group, CD4+, CD8+, FOXP3+TIL, and PD-L1+ immune cell infiltration was significantly higher in CXCL10-positive tumors than in CXCL10-negative tumors (*p* = 0.001, *p* = 0.001, *p* < 0.001 and *p* < 0.001, respectively). In HR-positive subgroup, similar to the whole group, CD4+, CD8+, FOXP3+TIL, and PD-L1+ immune cell infiltration was greater in CXCL10-positive tumors (all *p* < 0.05). In HR-negative subgroup, only FOXP3+TILs showed a significant difference between CXCL10-positive and negative groups (*p* = 0.001). CD4+ and CD8+ TILs tended to be higher in CXCL10-positive tumors. Although the number of cases was limited, PD-L1+ immune cell infiltration tended to be also high in CXCL10-positive tumors.

**Table 2:** Comparison of immune cell subset infiltration in relation to CXCL10 expression in DCIS and invasive carcinoma.

Immune cell subset	DCIS		*p* value	Invasive carcinoma		*p* value
	CXCL10 (+)	CXCL10 (−)		CXCL10 (+)	CXCL10 (−)	
**Total**						
CD4+ TIL	77.33 (23.50–116.67)	25.1 (7.00–52.75)	0.001	72.50 (35.25–155.50)	88.00 (47.00–186.00)	0.507
CD8+ TIL	36.33 (16.33–66.83)	12.33 (5.50–25.67)	0.001	120.00 (56.75–249.00)	65.00 (32.50–142.00)	0.008
FOXP3+TIL	4.67 (0.00–12.83)	0.00 (0.00–2.00)	< 0.001	13.00 (5.00–21.00)	5.00 (2.00–12.50)	0.001
PD-L1+ IC	11/21 (52.4)	34/200 (17.0)	< 0.001	24/36 (66.7)	40/113 (35.4)	0.001
**HR + subgroup**						
CD4+ TIL	58.83 (21.42 -104.58)	18.00 (5.00–47.08)	0.004	91.00 (38.75–160.25)	88.00 (49.00–172.00)	0.890
CD8+ TIL	31.67 (13.91–52.08)	11.33 (5.00–22.00)	0.006	70.00 (56.00–206.00)	58.00 (30.00–130.00)	0.120
FOXP3+TIL	0.50 (0.00–5.33)	0.00 (0.00–0.00)	0.024	7.00 (3.50–18.50)	5.00 (2.00–11.00)	0.179
PD-L1+ IC	6/14 (42.9)	20/156 (12.8)	0.009	7/18 (38.9)	29/62 (31.9)	0.563
**HR- subgroup**						
CD4+ TIL	109.33 (23.33–126.00)	50.17 (24.58–96.33)	0.175	70.00 (33.75–157.00)	106.00 (32.25–228.75)	0.563
CD8+ TIL	49.0 (31.00–76.33)	20.17 (8.25–51.75)	0.124	133.50 (89.00–322.50)	109.00 (36.00–262.00)	0.262
FOXP3+TIL	15.00 (8.00–17.33)	2.50 (0.00–7.75)	0.001	16.50 (10.75–31.50)	8.00 (3.75–15.00)	0.019
PD-L1+ IC	5/7 (71.4)	14/44 (31.8)	0.087	17/18 (94.4)	11/22 (50.0)	0.002

*P* values are calculated by Mann–Whitney *U* test.

Data are shown in are presented as median (interquartile range) for CD4+, CD8+, and FOXP3+ tumor infiltrating lymphocyte (TIL) subsets and frequency (%) for PD-L1+ immune cell (IC).

In invasive carcinoma (Table 2), CD8+ and FOXP3+TIL infiltration as well as PD-L1+ immune cell infiltration was higher in CXCL10-positive tumors (*p* = 0.007, *p* = 0.001, and *p* = 0.001, respectively). In HR-positive invasive carcinoma, there was no significant difference in CD4+, CD8+, FOXP3+TIL, and PD-L1+ immune cell infiltration according to CXCL10 expression. In HR-negative invasive carcinoma, FOXP3+TIL and PD-L1+ immune cell infiltration was also significantly higher in CXCL10-positive tumors than in CXCL10-negative tumors (*p* = 0.019 and *p* = 0.002, respectively).

## Discussion

Based on the findings of our previous study that TIL subset and PD-L1+ immune cell infiltration differ between DCIS and invasive carcinoma^7^, we further analyzed the difference in immune-related gene expression between DCIS and invasive carcinoma using Nanostring nCounter platform. Despite the limited number of cases, the immune-related gene that showed the most striking difference between the two disease groups was CXCL10. Therefore, using CXCL10 as a target molecule, we analyzed the role of CXCL10 in the progression of DCIS to invasive carcinoma.

CXCL9, CXCL10, CXCL11/CXCR3 axis is known to regulate differentiation of naïve T cells to T helper cells, and it activates and recruits immune cells such as CTLs, NK cells, NKT cells, and macrophages in response to IFN-γ^5,6,8^. CXCL10, which is a ligand of CXCR3, is mainly secreted by monocytes, endothelial cells, fibroblasts, and cancer cells^9^. The classic view on CXCL10 is that it prevents cancer through paracrine signaling as CXCL10 plays an important role in the recruitment and activation of immune cells^10^. However, there is increasing evidence that the CXCL9, CXCL10, CXCL11/CXCR3 axis plays a tumorigenic role causing tumor progression and metastasis both in vitro and in vivo; it is thought to occur via autocrine signaling of cancer cells which increases cell proliferation, angiogenesis, and metastasis^11–16^. In breast cancer, CXCL10 has been found to be overexpressed in tumors^11,17^, and secretion of CXCL10 by breast cancer cells has been demonstrated in vitro^18^. Our study showed increased proliferation and migration of both MCF-7 and MDA-MB-231 breast cancer cell lines with an increased dose of exogenous CXCL10. This concurs with the result of a previous study that CXCL10 not only acts to tumor microenvironment through paracrine signaling but also in an autocrine manner, giving a self-signal for proliferation and migration^18^.

EMT is a complex process, and through changes in its regulatory pathway such as loss of cellular adhesion, cell migration, and flow through the vascular system, it eventually leads to tumor metastasis^19^. It is characterized by decreased expression of E-cadherin and increased expression of mesenchymal markers. In breast cancer, molecular subtyping showed that luminal and HER2-enriched cancers retain more epithelial phenotype while triple negative or basal like cancers showed more mesenchymal features^20,21^. Our result showed decreased expression of E-cadherin in MCF-7 with an increased dose of CXCL10 with no expression of mesenchymal markers. Similarly, MDA-MB-231 showed decreased expression of mesenchymal markers with an increased dose of CXCL10 while there was no expression of E-cadherin at all. According to the study by Ren et al., in hepatocellular carcinoma (HCC), CXCL10 has been reported to accelerate EMT of HCC cells; the epithelial marker (E-cadherin) was up-regulated while mesenchymal cell markers (N-cadherin, fibronectin and vimentin) were downregulated when CXCL10 was silenced; they witnessed exact opposite results of over-expression of CXCL10^22^.

In the present study, CXCL10 expression was significantly higher in invasive carcinoma than in DCIS. In a previous study which evaluated expression of CXCL10 in 6 cases of breast cancer (comprised of 3 cases of DCIS and 3 cases of invasive carcinoma) compared to normal breast using immunohistochemistry, invasive carcinoma showed markedly increased expression of CXCL10. DCIS also showed increased expression compared to normal breast tissue; however, the intensity and distribution of staining was less compared to invasive carcinoma^23^. Ejaeidi and his colleagues showed in their study the elevation of CXCL10 in breast cancer patient’s sera compared to healthy controls in a hormone-independent manner^15^. Interestingly, in this study, CXCL10 expression was significantly different between DCIS and invasive carcinoma in HR-negative subgroup, but not in HR-positive subgroup. It can be postulated that since CXCL10 expression correlates with immune cell infiltration, and HR-negative tumors are more immunogenic than HR-positive tumors, the difference in CXCL10 expression in DCIS and invasive carcinoma may be accentuated in HR-negative tumors. Moreover, CXCL10 expression was significantly increased in DCIS-INV compared to DCIS in the whole group and in HR-negative subgroup, and there was no difference in CXCL10 expression between DCIS and invasive components within the same tumor. Ma et al. suggested in their study that gene expression alteration conferring the potential for invasive growth is already present in the pre-invasive stage^13^. The fact that CXCL10 expression does not differ between the DCIS and invasive components of the same tumor may represent early alteration of gene expression. However, as the number of cases used for comparison were limited, further confirmative analyses would be necessary using a large cohort.

TIL infiltration had a positive correlation with CXCL10 expression in both DCIS and invasive carcinoma in this study. Considering the paracrine effects of CXCL10 in immune cell migration, differentiation, and activation, areas with CXCL10 expression should have increased TIL infiltration. In DCIS, all subsets of TIL and PD-L1+ immune cells infiltration correlated with CXCL10 positivity while in invasive carcinoma, CD8+ and FOXP3+TIL, and PD-L1+ immune cell infiltration was significantly increased in CXCL10-positive tumors with no significant difference in CD4+ TIL infiltration. CXCR3, which is an inflammatory chemokine receptor of CXCL10, is known to be associated with CD4+ Th1 cells and CD8+ CTLs ^24,25^. These receptors are activated when their ligands CXCL10, CXCL9, and CXCL11 bind to the receptor. However, the reason why CD4+ TIL infiltration differs in DCIS but not in invasive carcinoma in relation to CXCL10 expression needs further investigation.

FOXP3+TIL infiltration increased in conjunction with CXCL10 expression. In a study on liver graft injury and tumor recurrence after liver transplantation, CXCL10/CXCR3 signaling upregulated at liver graft injury induced mobilization and recruitment of Tregs, which further promoted tumor recurrence after transplantation^26^. In pancreatic ductal adenocarcinoma, CXCL10 has been shown to recruit CD4+, CD8+, and CXCR3+ T cells as well as FOXP3+ Tregs^27^. In addition, there was a study which reported that CXCL10 drove increased transcription of T-bet and RORγ, leading polarization of naïve T cells to FOXP3- type 1 regulatory T cells or T helper 17 cells through STAT1, STAT4, and STAT5 phosphorylation^28^. While the exact functions of CXCL10 on FOXP3+ Tregs in breast cancer remain yet to be elucidated, it can be concluded that CXCL10 expression is associated with FOXP3+TIL infiltration in both DCIS and invasive carcinoma. Increased number of PD-L1+ immune cell infiltration was associated with CXCL10 expression in both DCIS and invasive carcinoma in this study. In line with our study results, CXCL9, CXCL10, CXCL11/CXCR3 axis has been suggested to regulate PD-L1 expression through STAT and PI3K-Akt pathways in gastric cancer^29^.

The current study included a relatively large number of cases that can provide a general idea of CXCL10 in tumorigenesis and its relationship with immune cell infiltration. Moreover, this is the first large study comparing CXCL10 expression in DCIS and invasive carcinoma. However, this study has some limitations. First, we did not show the mechanism by which CXCL10 promotes breast cancer cell proliferation and migration. Moreover, we did not show the effect of CXCL10 on tumor cell invasion directly, as wound healing assay measures the ability of the cells to migrate, not its ability to invade. Second, the role of CXCL10 can be best described when explained together with other chemokines such as CXCL9, CXCL10, CXCL11, and CXCR3. Especially, CXCR3, also known as GPCR9 or CD183, has three variants: CXCR3A, CXCR3B, and CXCR3-alt. These variants are known to have different functions with CXCR3A exerting a pro-tumor effect and CXCR3B an anti-tumor effect^30,31^. Since CXCL10 can have different effects depending on the binding receptor^32^, interpretation of CXC10 expression with CXCR3 expression may be useful. However, immunohistochemistry cannot differentiate the variants of CXCR3, and thus, variant-specific expression should be confirmed using a different modality. Furthermore, in this study, we did not compare the expression CXCL10 protein separately in tumor cells and composites of tumor microenvironment. In further studies, comparing such expression separately using multiplex immunohistochemistry in whole tumor sections would provide a better understanding the role of CXCL10 in tumor progression. Finally, evaluation of the association of CXCL10 with immune cell infiltration was confined to CD4+, CD8+, FOXP3+TILs, and PD-L1+ immune cells. Further investigations on the other immune cell subsets’ infiltration would be needed.

In conclusion, our study showed that among 770 immune related genes, CXCL10 revealed the highest difference in expression between DCIS and invasive carcinoma. We showed that CXCL10 induced increased cell proliferation and migration in MCF-7 and MDA-MB-231. Furthermore, through western blot assay, we showed that CXCL10 treatment led to a decrease in E-cadherin expression in MCF-7 and an increase in mesenchymal marker expression in MDA-MB-231, suggesting the possible role of CXCL10 as a modulator of epithelial-mesenchymal transition. CXCL10 mRNA and protein expression was significantly higher in invasive carcinoma than in DCIS in the whole group and HR-negative tumors. CXCL10 mRNA expression was also different between DCIS and DCIS-INV with increased expression of CXCL10 in DCIS-INV in the whole group and HR-negative group. In general, CXCL10 positive tumors showed higher infiltrations of CD4+, CD8+, and FOXP3+TILs and PD-L1+ immune cells. Taken together, CXCL10 seems to induce tumor cell proliferation, migration, and immune cell infiltration, suggesting its critical role in the progression of DCIS to invasive carcinoma.

## Materials and methods

### Cell culture and culture condition

Human breast cancer cell lines (MCF-7 and MDA-MB-231) were purchased from the Korean Cell Line Bank (Seoul, South Korea), and they were cultivated in RPMI 1640 media supplemented with 10% fetal bovine serum, 1% penicillin, and streptomycin at 37ºC in a humidified incubator with 5% CO_2._ RPMI 1640 medium, fetal bovine serum, phosphate buffered saline (PBS), and penincillin-streptomycin were obtained from Gibco, USA. Recombinant human CXCL10 was purchased from R&D systems (Minneapolis, MN).

### Cell proliferation assay

MCF-7 and MDA-MB-231 were seeded in a 96-well plate with 1 × 10^4^ cells per well for MCF-7 and 5 × 10^3^ cells per well for MDA-MB-231, respectively. After cell attachment, serial concentration gradients of CXCL10 were added to the wells, with three repeats for each concentration. Cell Counting Kit-8 (Dojindo Laboratories, Kimamoto, Japan) was used according to the manufacturer’s instructions to detect cell viability after 24 and 48 h. Cell proliferation was determined by comparing optical density at a wavelength of 450 nm using a microplate reader (BioTek instruments, Winooski, VT) by comparing the sample to the standard curve. Triplicate independent experiments were performed.

### Wound-healing assay

MCF-7 and MDA-MB-231 were seeded in a 6-well plate. When the cells were grown to at least 90% of confluency, a wound was made in the middle of the culture plate using SPLScar scratcher (SPL life sciences, Seoul, South Korea). Then the medium was replaced with fresh serum-free medium for the control well or with medium containing 40 ng/mL and 60 ng/mL of CXCL10. The images of the wounded areas were captured under the light microscope at 40× after 24 and 48 h. The area of the wound was quantified by Java’s Image J software (http://rsb.info.nih.gov). The areas of the wound after 24 h and 48 h were measured and compared to the control.

### Western blot

MCF-7 and MDA-MB-231 breast cell lines were seeded onto 100 mm dishes then cultured in RPMI1640 media treated with recombinant CXCL10 for 24 and 48 h. Cells were washed rapidly with ice-cold PBS and lysed in 1X RIPA lysis buffer (Cell signaling Technology, Danvers, MA, USA) according to the manufacturer’s instruction. The protein concentration of the supernatant was measured using a BCA reagent (Pierce, Rockford, IL, USA). For each sample, equal amounts of protein were denatured and fractionated by 10% (w/v) sodium dodecyl sulfate–polyacrylamide gel electrophoresis and transferred onto PVDF membrane. The membranes were then incubated overnight at 4 °C with antibodies of E-cadherin, β-catenin, N-cadherin, Zeb1, β-actin (Cell Signaling Technology), and vimentin (Santa Cruz Biotechnology, Paso Robles, CA, USA). After washing with Tris-buffered saline three times, the membranes were treated with horseradish peroxidase (HRP)-conjugated secondary antibodies (Cell signaling Technology) for 1 h. The signals were visualized using ECL reagents (Amersham Pharmacia Biotechnology, Buckinghamshire, UK) on an x-ray film (AGFA, Mortsel, Belgium).

### Tissue samples

Breast cancers that had been resected between 2003 and 2012 at Seoul National University Bundang Hospital were selected for this study. All tissue samples were surgically resected specimen from patients with primary breast cancer. Three sets of tumor samples were used, and the samples for DCIS included both pure DCIS and DCIS with microinvasion. In the first and second sets, cases resected after neoadjuvant chemotherapy were excluded. In the third set, 32 cases (8.6%) of invasive carcinoma treated by neoadjuvant chemotherapy before surgery were included. The first set was 48 cases of breast cancer samples (16 cases of DCIS and 32 cases of invasive carcinoma) chosen for Nanostring nCounter assay. We intentionally selected HR-positive and HR-negative samples in half. The second set was 120 cases of breast cancer (60 cases of DCIS and 60 cases of invasive carcinoma), which was used for validation by real time PCR and correlation of CXCL10 mRNA expression with TIL density in the tumors. The half of the samples in each group were HR-positive. Of the 60 invasive carcinoma cases, 24 cases which had a sufficient amount of DCIS component were selected for comparative analysis of the invasive and DCIS components within the same tumor. On H&E-stained sections, the average percentage of TILs in the stromal compartment was evaluated using 10% increments. In DCIS, the stromal compartment was defined according to a proposal from Immuno-Oncology Biomarker Working Group^33,34^. The third set was 593 cases of breast cancer composed of 223 cases of DCIS and 372 cases of invasive carcinoma. Clinicopathologic characteristics of DCIS and invasive carcinoma are presented in Supplementary Table S5 and S6. This set was used to evaluate the relationship between CXCL10 expression and clinicopathologic features of the tumors. Data on immune cell subset infiltration including CD4+, CD8+, and FOXP3+TILs, and PD-L1+ immune cells were from the previous studies^7,35^. After excluding missing values, a total of 223 cases of DCIS and 151 cases of invasive carcinoma were used for the comparison of immune cell subset infiltration in relation to CXCL10 expression.

Clinicopathologic information was obtained by reviewing the medical records and hematoxylin and eosin-stained sections. Expression of the basic biomarkers including estrogen receptor, progesterone receptor, HER2, p53, and Ki-67 was evaluated from the surgical specimens at the time of diagnosis using the same antibodies and interpretation criteria as in the previous studies^7,35^. This study was approved by the institutional review board (IRB) of Seoul National University Bundang Hospital (SNUBH) (IRB No B-1803/450-305), and informed consent was waived by the IRB of SNUBH. All experiments and procedures performed in studies involving human participants were in accordance with the ethical standards of the institutional research committee and with the 1964 Helsinki declaration and its later amendments or comparable ethical standards.

### Immune profiling using Nanostring nCounter assay

Using 10 μm thick sections of formalin-fixed paraffin-embedded (FFPE) tissue, RNA was extracted from tumor areas including tumor cells and tumor stromal components and comprising more than 70% of tumor cells using RecoverAll Total Nucleic Acid Isolation Kit (Ambion, Grand Island, NY, USA). The concentration of extracted RNA was determined using DS-11 Spectrophotometer (Denovix INC, Wilmington, DE, USA), and RNA quality check was done using Fragment Analyzer (Advanced Analytical Technologies, Ankeny, IA, USA). Of the 48 cases, one case with a low RNA concentration and low binding density was excluded from final analysis. A digital multiplexed NanoString nCounter human mRNA expression assay (NanoString Technologies, Seattle, WA, USA) was performed with nCounter PanCancer Immune Profiling Panel Kit that includes 770 immune-related gene and control genes, according to the manufacturer’s protocol. Target molecules were quantified by nCounter Digital Analyzer by counting the individual fluorescent barcodes. For each assay, a high-density scan encompassing 280 fields of view was performed. The data was collected using the nCounter Digital Analyzer after taking images of the immobilized fluorescent reporters in the sample cartilage with a CCD camera.

### Real-time quantitative PCR

In order to ensure tumor purity, we selected representative paraffin blocks with at least 70% tumor cells. Tumor areas in serial sections were marked manually by confirming the tumor area on H&E slides. Then, tumor area was macro-dissected in all cases. Total RNA was extracted using RNeasy FFPE Kit (Qiagen, Hilden, Germany) according to the manufacturer’s instructions. High capacity RNA-to-cDNA kit (Applied Biosystems, Foster City, CA, USA) protocol was used to transcribe total RNA into single-stranded cDNA. For real-time PCR, we used TaqMan Gene Expression Assay for both CXCL10 and the human glyceraldehyde-3-phostphate dehydrogenase (GAPDH) with TaqMan Universal PCR Mastermix (Applied Biosystems). Real-time PCR was performed using a StepOne Real-Time PCR systems (Applied Biosystems). The reaction was incubated at 95 °C for 10 min, followed by 50 cycles at 95 °C for 15 s, 60 °C for 1 min. The GAPDH was used in each plate as control.

CXCL10 mRNA expression was calculated using comparative Ct method (ΔCt). The threshold cycle (Ct) of CXCL10 was measured, and the data were normalized by subtracting the Ct value of an endogenous reference, GAPDH. For comparison of ΔCt value of mRNA of breast cancer with that of normal breast tissue, normalized ΔCt values were measured from 15 normal breast tissue samples excised for reduction mammoplasty. The average ΔCt value of benign breast tissue was used to calculate normal ΔCt value, and the average of this value was subtracted from ΔCt of CXCL10 treated samples to determine differences (ΔΔCt) and fold change (2^−ΔΔct^). For data with a sufficient amount of RNA and Ct value for the housekeeping gene but non-detectable Ct value for CXCL10, after repeated non-detection of the samples, the maximum number of PCR cycle, 50, was used as Ct value of each sample.

### Immunohistochemistry and scoring of CXCL10

Representative sections of each case were constructed into a tissue microarray as aforementioned. Immunohistochemical staining for CXCL10 was performed on tissue microarrays after staining optimization using positive and negative control and serial dilution. The submitted slides were deparaffinized and rehydrated in graded ethanol. Antigen retrieval was performed by immersing the slides in citrate buffer (pH 6.0) for 30 min in the steamer. Using a 3% H_2_O_2_-methanol solution, endogenous peroxidase activity was blocked, after which the slides were incubated in 10% normal goat serum for 30 min to prevent nonspecific staining. Using anti-IP10 antibody (CXCL10) (ab9807, polyclonal; 1:500 dilution; Abcam), the slides were incubated for 1 h at room temperature. Then, the sections were incubated with a HRP-labeled polymer conjugated with secondary antibodies (DAKO Envision detection kit, Dako) for 30 min. Diaminobenzidine was used as a chromogen, and the sections were counterstained with Mayer’s hematoxylin.

Expression of CXCL10 in the tumor area including tumor cells, immune cells, and stromal cells were evaluated without knowledge of the clinicopathologic information. Positively-stained tumor area with a dot-like cytoplasmic or membranous staining pattern was considered positive regardless of staining intensity. CXCL10 was considered to be positive when at least 1% of the tumor area was positively stained.

### Statistical analysis

In immune profiling using Nanostring nCounter assay, analysis of raw mRNA data was performed using NanoString technologies nSolver analysis software version 4.0. The mRNA expression data was normalized using housekeeping genes. R software was used for comparison of mRNA expression between two groups. Difference in gene expression between DCIS and invasive carcinoma was presented as a Log2 fold change, and *p* values were adjusted by Benjamini-Yekutieli procedure. Other statistical analysis was performed using Statistical package, SPSS version 25.0 for Windows (IBM Corp., Armonk, NY). Mann–Whitney *U* test was performed for statistical analysis for cell proliferation and wound-healing assays. For Real-time PCR of CXCL10, the fold changes (2^−ΔΔCt^) of all groups including DCIS, invasive carcinoma, and (DCIS-INV, did not show a normal distribution and thus, Mann–Whitney *U* test was used for analysis. For comparison of CXCL10 mRNA expression between invasive component and in situ components within the same tumor, Wilcoxon signed rank test was used. In order to evaluate the correlation between CXCL10 mRNA expression and TILs in both DCIS and invasive carcinoma, Spearman’s rank correlation test was used. Chi-square and Fisher’s exact tests were used to evaluate CXCL10 protein expression in relation to clinicopathologic features of DCIS and invasive carcinoma. Mann–Whitney *U* test were used to analyze CD4+, CD8+, and FOXP3+TILs and PD-L1+ immune cell infiltration in relation to CXCL10 protein expression. *P* values less than 0.05 were considered significant with all reported *p* values being two-sided.

## Supplementary Information

Supplementary Figure S1.

Supplementary Tables.

## Data Availability

The datasets used and/or analyzed during the current study are available from the corresponding author on reasonable request.
