# Supplementary material for: Role of CXCL10 in the progression of in situ to invasive carcinoma of the breast
Source: Sci Rep. 2021 Sep 9;11:18007. doi: 10.1038/s41598-021-97390-5 (PMC8429587; doi:10.1038/s41598-021-97390-5)

MCF-7 cells (24 hours) - Western Blot

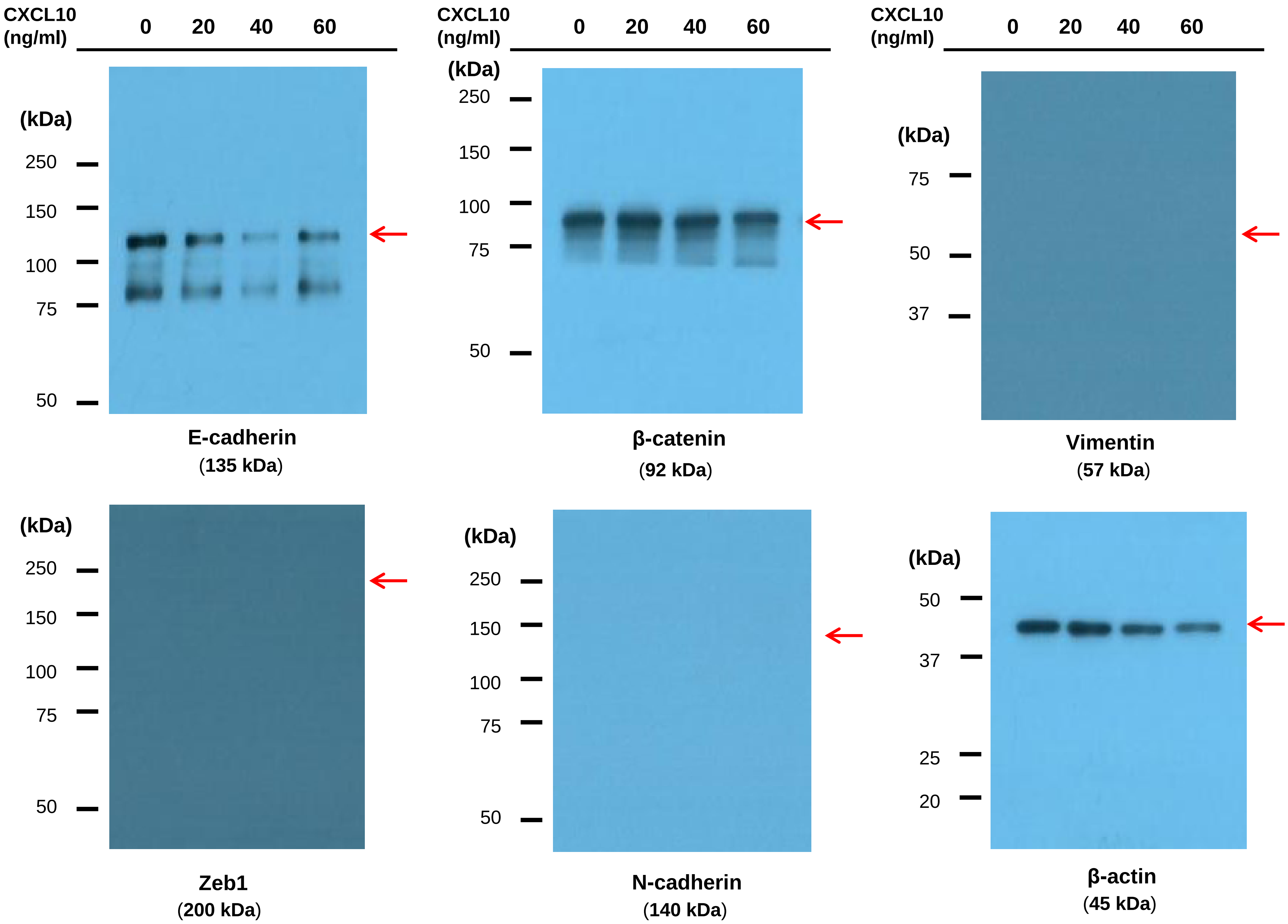

MCF-7 cells (48 hours) - Western Blot

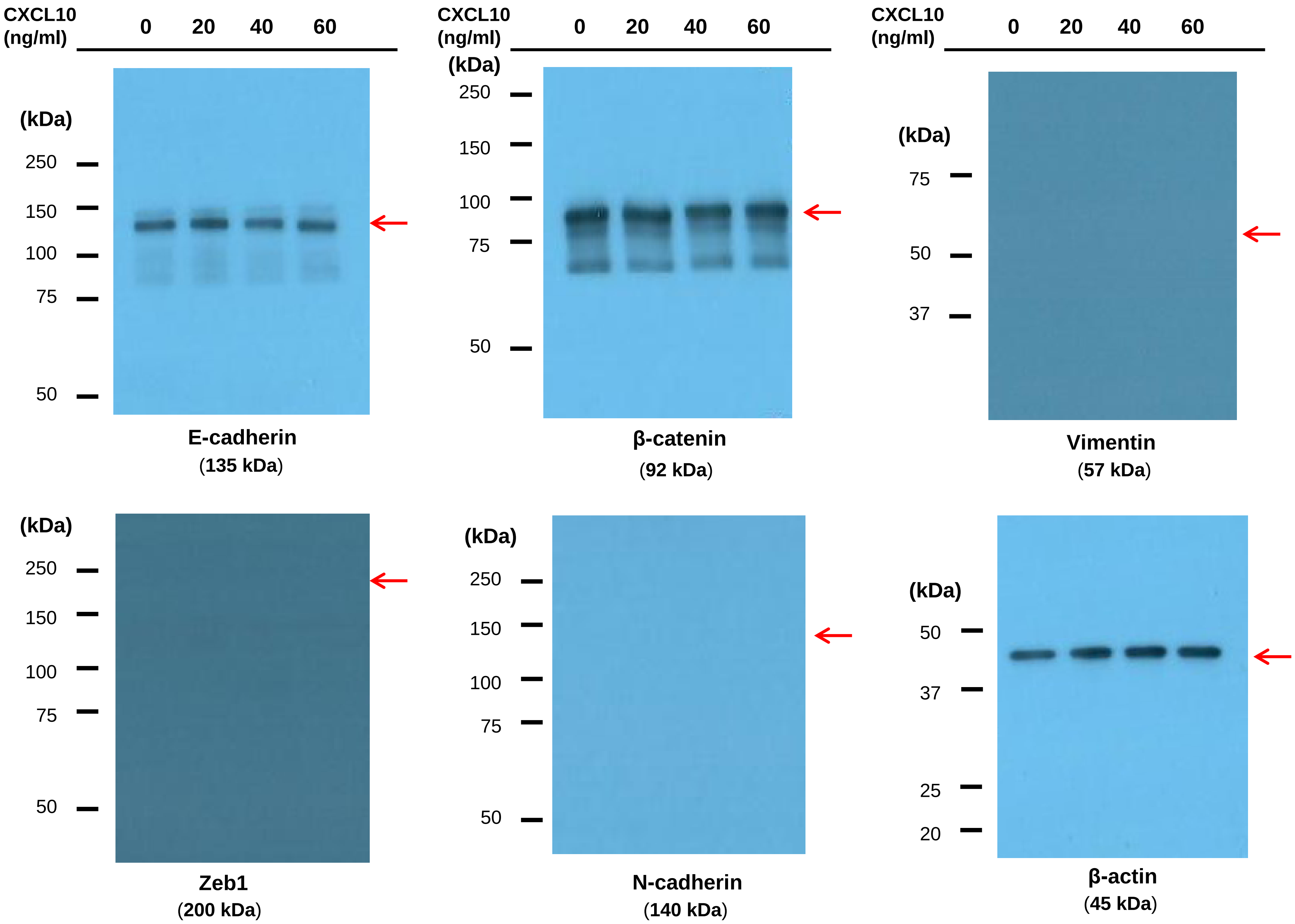

MDA-MB-231 cells (24 hours) - Western Blot

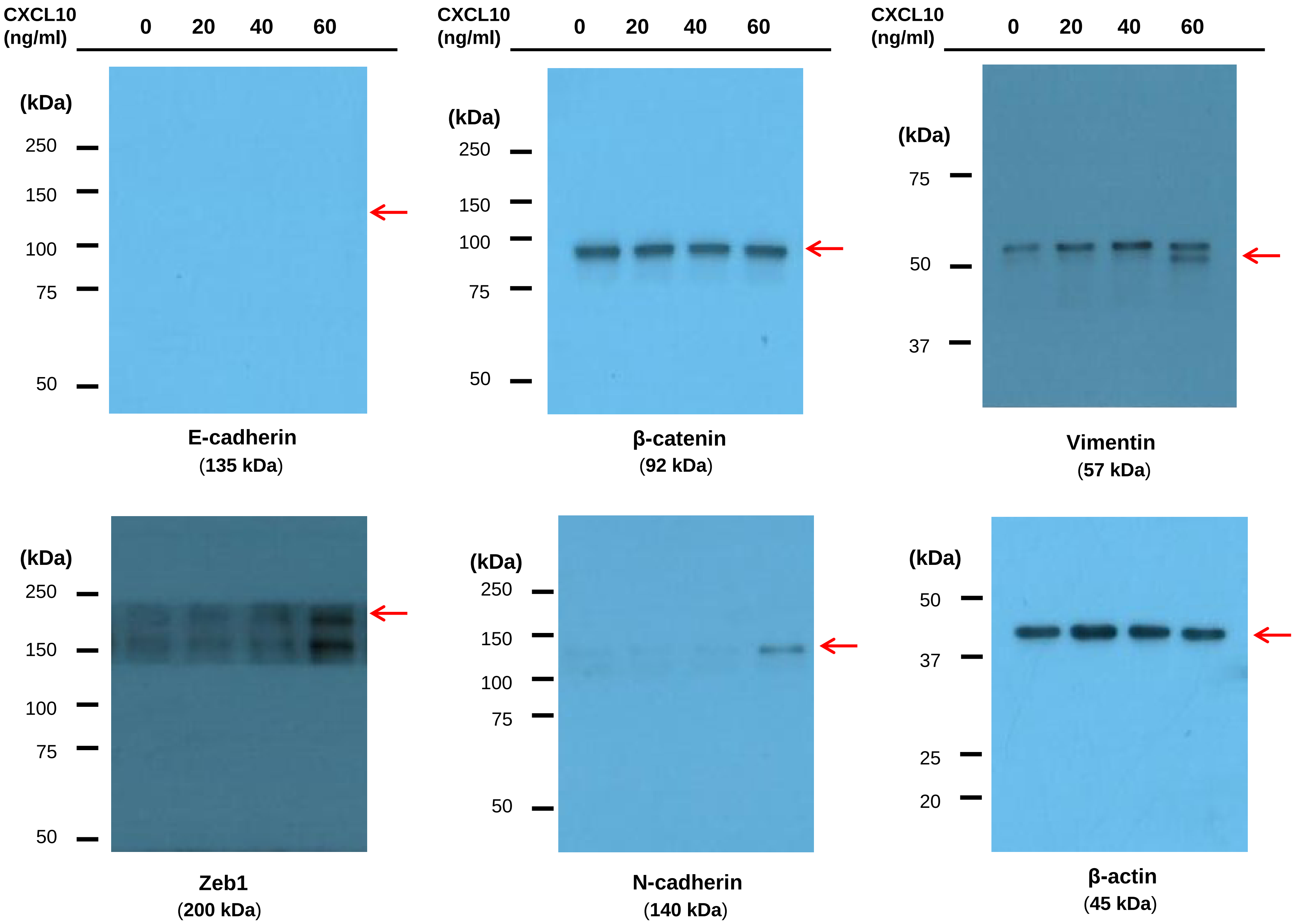

MDA-MB-231 cells (48 hours) - Western Blot

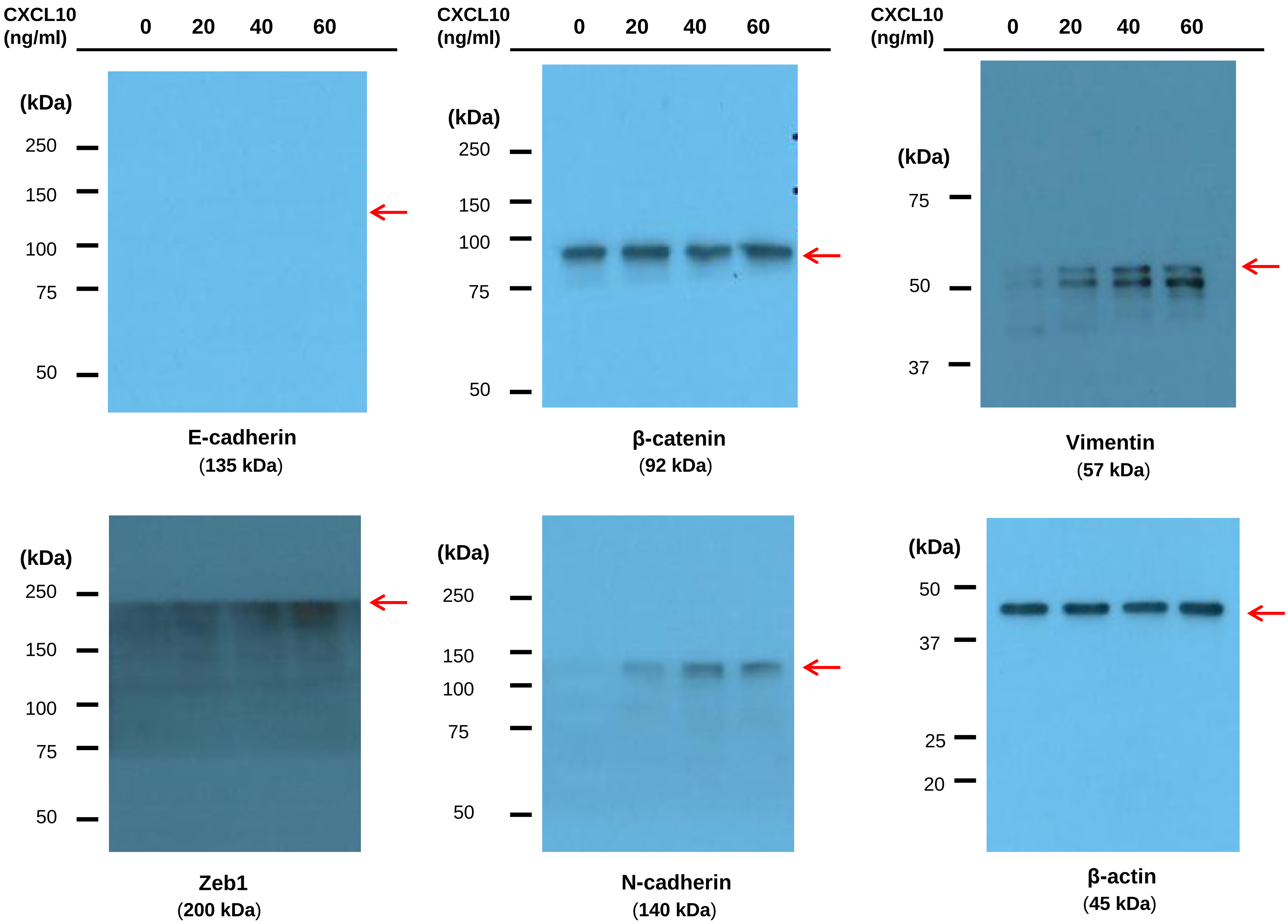

Supplement: Supplementary file 1 — Supplementary Figure S1. [file 41598_2021_97390_MOESM1_ESM.pdf]
