# Supplementary material for: Role of CXCL10 in the progression of in situ to invasive carcinoma of the breast
Source: Sci Rep. 2021 Sep 9;11:18007. doi: 10.1038/s41598-021-97390-5 (PMC8429587; doi:10.1038/s41598-021-97390-5)
Supplement: Supplementary file 2 — Supplementary Tables. [file 41598_2021_97390_MOESM2_ESM.docx]

**Supplementary Table S1. List of top 20 genes with a significant fold change between DCIS and invasive carcinoma in hormone receptor-positive tumors**

| **Gene** | **Log2 fold change** | ***p* value** | **Adjusted *p* value** |
| --- | --- | --- | --- |
| S100A8 | 2.25 | 0.00714 | 1.000 |
| LAG3 | 2.03 | 0.00368 | 1.000 |
| CXCL10 | 1.94 | 0.00090 | 0.727 |
| CXCL9 | 1.90 | 0.00463 | 1.000 |
| BIRC5 | 1.88 | 0.00035 | 0.727 |
| PLAU | 1.45 | 0.00116 | 0.798 |
| CDK1 | 1.38 | 0.00160 | 0.965 |
| HLA-DRA | 1.30 | 0.00922 | 1.000 |
| TFRC | 1.21 | 0.00071 | 0.727 |
| CXCR4 | 1.00 | 0.00823 | 1.000 |
| F12 | 0.99 | 0.00382 | 1.000 |
| GTF3C1 | 0.91 | 0.00193 | 1.000 |
| BAX | 0.40 | 0.00335 | 1.000 |
| CFI | -0.67 | 0.00584 | 1.000 |
| IL6R | -0.87 | 0.00322 | 1.000 |
| MAP2K4 | -0.93 | 0.00088 | 0.727 |
| CCL18 | -1.36 | 0.00634 | 1.000 |
| CXCL1 | -1.48 | 0.00723 | 1.000 |
| CXCL2 | -1.69 | 0.00075 | 0.727 |
| LTF | -2.46 | 0.00069 | 0.727 |

*p-*values were adjusted by Benjamini-Yekutieli procedure.

**Supplementary Table S2. List of top 20 genes with a significant fold change between DCIS and invasive carcinoma in hormone receptor-negative tumors**

| **Gene** | **Log2 fold change** | ***p* value** | **Adjusted *p* value** |
| --- | --- | --- | --- |
| CXCL10 | 3.44 | 0.00019 | 0.570 |
| HLA-A | 2.72 | 0.00429 | 1.000 |
| IFITM1 | 2.61 | 0.00092 | 1.000 |
| CXCL9 | 2.56 | 0.00033 | 0.570 |
| APOE | 2.04 | 0.00443 | 1.000 |
| COL3A1 | 2.01 | 0.00518 | 1.000 |
| CTSS | 1.98 | 0.00035 | 0.570 |
| SERPING1 | 1.90 | 0.00141 | 1.000 |
| CCL5 | 1.89 | 0.00449 | 1.000 |
| LCP1 | 1.78 | 0.00173 | 1.000 |
| C1S | 1.74 | 0.00123 | 1.000 |
| PLAU | 1.32 | 0.00314 | 1.000 |
| PDGFRB | 1.14 | 0.00288 | 1.000 |
| GPI | 1.05 | 0.00523 | 1.000 |
| FCGR2A | 0.98 | 0.00281 | 1.000 |
| BMI1 | 0.94 | 0.00537 | 1.000 |
| IFNAR2 | 0.86 | 0.00260 | 1.000 |
| CD83 | 0.70 | 0.00366 | 1.000 |
| TRAF3 | 0.61 | 0.00187 | 1.000 |
| CSF3 | -2.82 | 0.00453 | 1.000 |

*p-*values were adjusted by Benjamini-Yekutieli procedure.

**Supplementary Table S3. Relationship between CXCL10 expression and clinicopathologic features of DCIS**

| **Clinicopathologic feature** | **CXCL10** | | ***p* value** |
| --- | --- | --- | --- |
|  | **Negative** | **Positive** |  |
| Extent |  |  | 0.722 |
| <3.8cm | 114 (56.4) | 11 (52.4) |  |
| ≥3.8cm | 88 (43.6) | 10 (47.6) |  |
| Nuclear grade |  |  | 0.441 |
| Low | 11 (5.4) | 0 (0) |  |
| Intermediate | 96 (47.5) | 9 (42.9) |  |
| High | 95 (47.0) | 12 (57.1) |  |
| Comedo-type necrosis |  |  | 0.399 |
| Absent | 134 (66.3) | 12 (57.1) |  |
| Present | 68 (33.7) | 9 (42.9) |  |
| Microinvasion |  |  | 0.453 |
| Present | 52 (25.7) | 7 (11.9) |  |
| Absent | 150 (74.3) | 14 (66.7) |  |
| ER |  |  | 0.589 |
| Positive | 156 (77.2) | 15 (71.4) |  |
| Negative | 46 (22.8) | 6 (28.6) |  |
| PR |  |  | 0.132 |
| Positive | 147 (72.8) | 12 (57.1) |  |
| Negative | 55 (27.2) | 9 (42.9) |  |
| HER2 status |  |  | 0.675 |
| Negative | 155 (76.7) | 15 (71.4) |  |
| Positive | 47 (23.3) | 6 (28.6) |  |
| Ki67 index |  |  | 0.824 |
| Low (<10%) | 130 (64.4) | 13 (61.9) |  |
| High (≥10%) | 72 (35.6) | 8 (38.1) |  |
| P53 overexpression |  |  | 0.548 |
| Absent | 167 (82.7) | 16 (76.2) |  |
| Present | 35 (17.3) | 5 (23.8) |  |
| Subtype |  |  | 0.487 |
| Luminal A | 124 (61.4) | 12 (57.1) |  |
| Luminal B | 34 (16.8) | 3 (14.3) |  |
| HER2+ | 25 (12.4) | 5 (23.8) |  |
| Triple negative | 19 (9.4) | 1 (4.8) |  |

*P* values are calculated by Chi-square or Fisher’s exact test. Number in parenthesis indicates percentage.

**Supplementary Table S4. Relationship between CXCL10 expression and clinicopathologic features of invasive carcinoma**

| **Clinicopathologic features** | **CXCL10** | | | ***p* value** |
| --- | --- | --- | --- | --- |
|  | **Negative** | **Positive** | |  |
| T stage |  | |  | 0.057 |
| T1 | 271 (93.1) | | 80 (98.8) |  |
| T2-T4 | 20 (6.9) | | 1 (1.2) |  |
| N stage |  | |  | 0.129 |
| N0 | 152 (52.2) | | 50 (24.8) |  |
| N1-N3 | 139 (47.8) | | 31 (38.3) |  |
| Histologic grade |  | |  | <0.001 |
| Low | 61 (21.0) | | 5 (6.2) |  |
| Intermediate | 107 (36.8) | | 17 (21.0) |  |
| High | 123 (42.3) | | 59 (72.8) |  |
| Lymphovascular invasion |  | |  | 0.551 |
| Absent | 158 (54.3) | | 47 (58.0) |  |
| Present | 133 (45.7) | | 34 (42.0) |  |
| ER |  | |  | <0.001 |
| Positive | 224 (77.0) | | 33 (40.7) |  |
| Negative | 67 (23.0) | | 48 (59.3) |  |
| PR |  | |  | <0.001 |
| Positive | 197 (67.7) | | 30 (37.0) |  |
| Negative | 94 (32.3) | | 51 (63.0) |  |
| HER2 status |  | |  | 0.144 |
| Negative | 237 (81.4) | | 60 (74.1) |  |
| Positive | 54 (18.6) | | 21 (25.9) |  |
| Ki67 index |  | |  | <0.001 |
| Low (<20%) | 188 (64.6) | | 26 (32.1) |  |
| High (≥20%) | 103 (35.4) | | 55 (67.9) |  |
| P53 overexpression |  | |  | 0.001 |
| Absent | 231 (79.4) | | 50 (61.7) |  |
| Present | 60 (20.6) | | 31 (38.3) |  |
| Subtype |  | |  | <0.001 |
| Luminal A | 155 (53.3) | | 22 (27.2) |  |
| Luminal B | 74 (25.4) | | 13 (16.0) |  |
| HER2+ | 20 (6.9) | | 15 (18.5) |  |
| Triple negative | 42 (14.4) | | 31(38.3)_ |  |

*P* values were calculated by Chi-square or Fisher’s exact test. Number in parenthesis indicates percentage.

**Supplementary Table S5. Clinicopathologic characteristics of DCIS in the third set**

| **Characteristic** | **No. (n=223)** |
| --- | --- |
| Age at diagnosis, years |  |
| Mean ± standard deviation | 48.6 ± 10.3 |
| Extent, cm |  |
| Mean ± standard deviation | 3.8 ± 2.5 |
| Nuclear grade |  |
| Low | 11 (4.9) |
| Intermediate | 105 (47.1) |
| High | 107 (48.0) |
| Comedo-type necrosis |  |
| Absent | 146 (65.5) |
| Present | 77 (34.5) |
| Microinvasion |  |
| Present | 59 (26.5) |
| Absent | 164 (73.5) |
| Estrogen receptor (1% ) |  |
| Positive | 171 (76.7) |
| Negative | 52 (23.3) |
| Progesterone receptor (1%) |  |
| Positive | 159 (71.3) |
| Negative | 64 (28.7) |
| HER2 status |  |
| Negative | 170 (76.2) |
| Positive | 53 (23.8) |
| Ki67 index |  |
| Low (<10%) | 143 (64.1) |
| High (≥10%) | 80 (35.9) |
| P53 overexpression |  |
| Absent | 183 (82.1) |
| Present | 40 (17.9) |
| Subtype |  |
| Luminal A | 136 (61.0) |
| Luminal B | 37 (16.6) |
| HER2+ | 30 (13.5) |
| Triple negative | 20 (9.0) |

Unless specified, numbers in parentheses indicate percentage.

**Supplementary Table S6. Clinicopathologic characteristics of invasive carcinomas in the third set**

| **Characteristic** | **No. (n=372)** |
| --- | --- |
| Age at diagnosis, years |  |
| Mean ± standard deviation | 50.5 ± 11.8 |
| T stage |  |
| T1 | 156 (41.9) |
| T2 | 195 (52.4) |
| T3 | 13 (3.5 |
| T4 | 8 (2.2) |
| N stage |  |
| N0 | 202 (54.3) |
| N1 | 102 (27.4) |
| N2 | 35 (9.4) |
| N3 | 33 (8.9) |
| Histologic subtype |  |
| Invasive carcinoma of no special type | 339 (91.1) |
| Mucinous carcinoma | 12 (3.2) |
| Metaplastic carcinoma | 10 (2.7) |
| Others | 11 (3.0) |
| Histologic grade |  |
| Low | 66 (17.7) |
| Intermediate | 124 (33.3) |
| High | 182 (48.9) |
| Lymphovascular invasion |  |
| Absent | 205 (55.1) |
| Present | 167 (44.9) |
| Estrogen receptor |  |
| Positive | 257 (69.1) |
| Negative | 115 (30.9) |
| Progesterone receptor |  |
| Positive | 227 (61.0) |
| Negative | 145 (39.0) |
| HER2 status |  |
| Negative | 297 (79.8) |
| Positive | 75 (20.2) |
| Ki67 index |  |
| Low (<20%) | 214 (57.5) |
| High (≥20%) | 158 (42.5) |
| P53 overexpression |  |
| Absent | 281 (75.5) |
| Present | 91 (24.5) |
| Subtype |  |
| Luminal A | 176 (47.3) |
| Luminal B | 81 (21.8) |
| HER2+ | 38 (10.2) |
| Triple negative | 77 (20.7) |

Unless specified, numbers in parentheses indicate percentages.
